# Darpp-32 and Its Truncated Variant t-Darpp Have Antagonistic Effects on Breast Cancer Cell Growth and Herceptin Resistance

**DOI:** 10.1371/journal.pone.0006220

**Published:** 2009-07-13

**Authors:** Long Gu, Sarah Waliany, Susan E. Kane

**Affiliations:** Division of Tumor Cell Biology, Beckman Research Institute of City of Hope, Duarte, California, United States of America; Roswell Park Cancer Institute, United States of America

## Abstract

**Background:**

Herceptin (trastuzumab) is a humanized monoclonal antibody that is approved for the treatment of metastatic breast cancer patients whose tumors overexpress Her2 (erbB2/neu). Up to 70% of Her2-positive breast cancers demonstrate a response to Herceptin-based therapies, but resistance almost inevitably arises within a year of the initial response. To help understand the mechanism of Herceptin resistance, we isolated clonal variants of Her2-positive BT474 human breast cancer cells (BT/Her^R^) that are highly resistant to Herceptin. These cell lines exhibit sustained PI3K/Akt signaling as an essential component of Herceptin-resistant proliferation. Several genes in the protein kinase A (PKA) signaling network have altered expression in BT/Her^R^ cells, including PPP1R1B, which encodes a 32 kDa protein known as Darpp-32 and its amino-terminal truncated variant, t-Darpp. The purpose of the current work was to determine the role of Darpp-32 and t-Darpp in Herceptin resistance.

**Methodology and Results:**

We determined expression of Darpp-32 and t-Darpp in BT/Her^R^ cells selected for resistance to Herceptin. Subsequently, cDNAs encoding the two isoforms of Darpp-32 were transfected, separately and together, into Her2-positive SK-Br-3 breast cancer cells. Transfected cells were tested for resistance to Herceptin and Herceptin-mediated dephosphorylation of Akt. DNA binding activity by the cAMP response element binding protein (CREB) was also measured. We found that BT/Her^R^ cells overexpressed t-Darpp but not Darpp-32. Moreover, t-Darpp overexpression in SK-Br-3 cells was sufficient for conferring resistance to Herceptin and Herceptin-mediated dephosphorylation of Akt. Darpp-32 co-expression reversed t-Darpp's effects on Herceptin resistance and Akt phosphorylation. t-Darpp overexpression led to increased CREB binding activity, which was also reversible by Darpp-32.

**Conclusions:**

t-Darpp and Darpp-32 appear to have antagonistic effects on Herceptin resistance. We present a unified model by which these effects might be mediated via the PKA regulatory network.

## Introduction

Her2, a member of the ErbB family of receptor tyrosine kinases, is overexpressed in about 25% of human breast cancers [1]. Herceptin (trastuzumab) is a humanized monoclonal antibody targeted to Her2 and approved for use against Her2-positive metastatic breast cancer [2]. Despite a fairly robust response rate to Herceptin-based therapies in these patients, resistance frequently arises within one year of an initial response [3]–[7]. The determinants of response or resistance to anti-cancer drugs are often complex. In the case of Herceptin, which works primarily by shutting down the PI3K/Akt signal transduction pathway, the key determinant of response appears to be the ability to modulate Akt phosphorylation. Failure to modulate phospho-Akt results in resistance [8]–[10].

Cells have several mechanisms by which to sustain Akt signaling in the face of Herceptin [9], [11], including mutation of *PIK3A*, the gene that codes for the p110α catalytic subunit of PI3K [12], [13]; mutation or deletion of *PTEN*, whose product is a dual lipid/protein phosphatase responsible for down-modulating phosphatidyl-inositol 3,4,5 triphosphate that is the product of PI3K [13]–[15]; up-regulation or activation of receptor tyrosine kinases such as EGFR, Her3, IGFR1 or MET to compensate for Her2 inhibition [16]–[20]; down-regulation of phosphatases that are responsible for dephosphorylating Akt as a negative feedback mechanism [21], [22]; and up-regulation of phosphatase inhibitors that modulate the effect of those phosphatases and thus dampen the negative feedback [23]. There are also ways of bypassing phospho-Akt altogether, either by promoting signal transduction at a point downstream of Akt or by activating a parallel pathway(s) that can compensate for loss of PI3K/Akt signaling [24]–[26].

Using BT/Her^R^1.0 cell lines selected *in vitro* for their resistance to 1 µM Herceptin [8], we have identified the protein kinase A (PKA) pathway as a possible central regulator of PI3K/Akt signaling and possible compensatory pathway for survival in the presence of Herceptin. In a separate report, we demonstrate that either stimulation of PKA with forskolin or down-regulation of the RIIα regulatory subunit of PKA with siRNA was sufficient for conferring partial resistance to Herceptin-mediated growth arrest and Akt dephosphorylation (L. Gu and S.E. Kane, manuscript submitted). Additional PKA-related gene expression changes observed in BT/Her^R^1.0 clones include down-regulation of the PKIγ gene, whose product acts as an endogenous inhibitor of PKA [27]; down-regulation of the gene that codes for PTG (protein targeting to glycogen), a scaffold protein [28] that promotes the activity of PP-1, a downstream target for negative regulation by PKA and itself a negative regulator of Akt; and up-regulation of the PPP1R1B gene, which codes for Darpp-32, a substrate for and feedback inhibitor of PKA and also an inhibitor of PP-1 [29]. The PPP1R1B locus also codes for t-Darpp, a transcriptional variant and amino-truncated isoform of Darpp-32 whose function within the PKA pathway is not known, but which is overexpressed in many adenocarcinomas and has been associated with drug resistance in cell lines [30]–[33].

We now demonstrate that it was t-Darpp, and not Darpp-32, that was overexpressed in BT/Her^R^ cells selected for Herceptin resistance and that transfection and overexpression of exogenous t-Darpp in Her2-positive SK-Br-3 cells was sufficient for conferring resistance to Herceptin and Herceptin-mediated dephosphorylation of Akt. Darpp-32 co-expression reversed t-Darpp's effects on Herceptin resistance and Akt phosphorylation. Overexpression of t-Darpp also led to increased CREB binding activity, which was also reversible by Darpp-32. We present a model by which the PKA pathway and its regulatory components might impact cellular response to Herceptin.

## Materials and Methods

### Cell culture

The human breast cancer cell lines BT474 and SK-Br-3 were obtained from the American Type Culture Collection (Rockville, MD). BT474 cells were maintained in DMEM with 10% FBS and 1% penicillin/streptomycin in 5% CO_2_. BT/Her^R^ clones, previously derived from BT474 cells after a six-month selection in the continuous presence of Herceptin [8], were maintained in the same culture conditions as the BT474 cells. They were regularly tested in Herceptin-containing medium to verify their drug resistance. SK-Br-3 cell clones were maintained in McCoy's Medium 5A with 10% FBS, 1% penicillin/streptomycin, and 1%L-glutamine in 5% CO_2_.

### Stable transfections

Darpp-32 and t-Darpp cDNAs were a kind gift from Wael El-Rifai. Darpp-32 was in the pcDNA3.1/Zeo vector. We subcloned the t-Darpp cDNA into the pcDNA3.0/Neo vector to remove a FLAG-tag present at the N-terminus of the received cDNA and restore its native sequence. Plasmid DNAs were transfected into SK-Br-3 cells or clones derived from SK-Br-3 cells by Lipofectamine 2000 according to manufacturer's instruction. SK-Br-3 cells transfected with a pcDNA3.0/Neo vector backbone were selected and maintained in the presence of 500 µg/ml G418. SK-Br-3 cell clones transfected with a pcDNA3.1/Zeocin vector backbone were selected and maintained in the presence of 200 µg/ml Zeocin.

### RNA preparation and real time RT-PCR

Total RNA was extracted and purified using the RNeasy kit purchased from Qiagen (Valencia, CA) and the integrity of the RNA was verified using the Agilent Technologies Bioanalyzer 2100 (Santa Clara, CA). cDNAs were synthesized by random priming, using purified total RNA as template. The t-Darpp and Darpp-32 mRNA levels were quantified by a SYBR^®^ Green assay, using a reaction mixture purchased from Applied Biosystems (Foster City, CA). Primers 5′-TCTGCCTCTCCCGTCCTTCTand 5′-AGAGACACACGCGGAGAGGAwere used for quantifying Darpp-32 mRNA and primers5′-TGAGGCTCAGGGACCCAAAG and 5′-CTGCAGCCTTACAGAGACTGG were used for quantifying t-Darpp mRNA. The PCR and real time quantification were carried out in an auto-lid dual 384-well GeneAmp® PCR System (Model 9700, Applied Biosystems). Quadruplicate measurements were made on a single isolation of RNA from each cell line analyzed. Statistical significances were determined by two-tailed t-test.

Raw microarray data referenced as part of a separate report are described in accordance with MIAME guidelines and are deposited in the Gene Expression Omnibus database, Accession No. GSE15043).

### Sulforhodamine B (SRB) assay

Cells were seeded into each well of 96-well tissue culture plates at a density of 4×10^3^/well and allowed to attach overnight. On day 0, medium with or without Herceptin was added. After growing in the presence or absence of Herceptin for 7 days, cells were fixed with 10% trichloroacetic acid and stained with 0.4% SRB (w/v) (Sigma, Saint Louis, MO) according to manufacturer's instructions. 10 nM Tris Base solution was used to solubilize the bound stain and absorbances at 565 nm were measured.

### Antibodies and Western analysis

A rabbit monoclonal antibody recognizing the N-terminal sequence that is unique to the Darpp-32 protein (#2302) and a rabbit monoclonal antibody recognizing only Akt/PKB that is phosphorylated on Ser473 (#4058) were purchased from Cell Signal Technologies (Danvers, MA). A rabbit polyclonal antibody recognizing both Darpp-32 and t-Darpp (H-62) and a rabbit polyclonal antibody recognizing total Akt/PKB (sc-8312) were purchased from Santa Cruz Biotechnology (Santa Cruz, CA). Cell samples were dissolved and sonicated in 2x Laemmli sample buffer. After boiling for five minutes, equal amounts of total protein, as determined by the *RC DC* Protein Assay kit purchased from Bio-Rad (Hercules, CA), were loaded onto a 10% SDS-polyacrylamide gel and separated proteins were transferred onto a nitrocellulose membrane. The membrane was blocked with 5% non-fat dry milk and incubated with primary antibody in the blocking buffer. After incubation with a peroxidase-conjugated anti-mouse IgG secondary antibody, the protein of interest was detected using an ECL kit purchased from GE HealthCare (Piscataway, NJ). For repeated antibody probing, the membrane was stripped with a Western blot stripping buffer purchased from Pierce (Rockford, IL).

### Electrophoretic mobility shift assay

Nuclear extracts were prepared using CelLytic™ NuCLEAR™ Extraction Kit (Sigma, Saint Louis, MO). Electrophoretic mobility shift assay (EMSA) was done using a gel shift assay kit purchased from Promega (Madison, WI) according to manufacturer's instructions. Briefly, double-stranded oligonucleotide containing a consensus CREB (cyclic AMP response element-binding protein) response element (CRE) was labeled with γ-^32^P ATP by end-labeling. DNA-protein binding reactions were performed by incubating 5 µg of nuclear protein with excess ^32^P-labeled CRE oligonucleotide in the buffer supplied with the assay kit. For competition binding, 1 pmol of an unlabeled CRE oligonucleotide or an unlabeled non-specific oligonucleotide was added. After incubation at room temperature for 20 min, binding reactions were resolved on a 4% native polyacrylamide gel. The gel was then dried onto Whatman paper and radioactivity was visualized by autoradiography. The autoradiography images were digitized by a high-resolution scanner and the densities of individual bands were measured by ImageQuant^MT^ 5.2 (GE Healthcare, Piscataway, NJ).

## Results

### Enhanced expression of t-Darpp in BT/Her^R^ cell clones

We previously reported on the isolation and initial characterization of BT/Her^R^1.0 cell lines selected for their resistance to Herceptin at 1.0 µM concentration [8]. A salient feature of BT/Her^R^1.0 cells is their sustained phospho-Akt levels and Akt activity in the presence of Herceptin. As part of a separate study, gene expression profiling revealed that the PPP1R1B gene was up-regulated by 9- to 50-fold in two independent BT/Her^R^1.0 clones (raw microarray data are deposited in the Gene Expression Omnibus database, Accession No. GSE15043).

**Figure 1:**
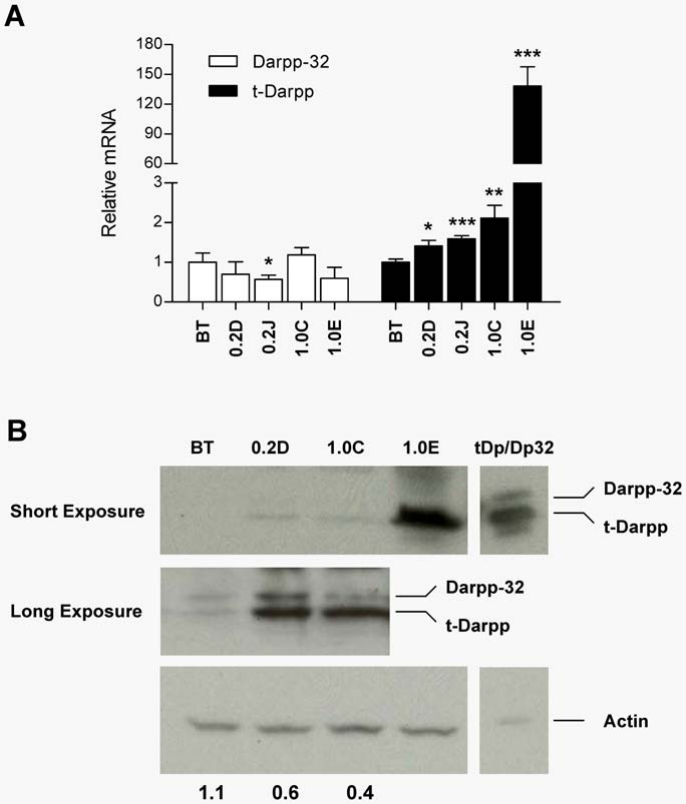
Expression of Darpp-32 and t-Darpp in BT/Her^R^ cells. (A) Expression of Darpp-32 (open bars) and t-Darpp (filled bars) mRNA in BT474 cells and BT/Her^R^ clones was analyzed by a SYBR Green assay. Shown are the average expression levels (±S.D.) of Darpp-32 and t-Darpp mRNAs in each indicated cell line relative to the corresponding mRNA levels in BT474 cells. Quadruplicate measurements were made on a single isolation of RNA from each cell line analyzed. Statistically significant differences from BT cells were determined by two-tailed t-test. *, p<0.05; **, p<0.01, ***, p<0.001. (B) Expression of Darpp-32 and t-Darpp proteins in BT474 and Her^R^/BT clones was analyzed by Western hybridization. SK-Br-3 cells exogenously expressing both t-Darpp and Darpp-32 (tDp/Dp32) were used as control for the respective forms of the protein; this lane was loaded with one-fifth as much lysate as the other lanes to account for their high exogenous t-Darpp/Darpp-32 expression. The top panel shows a short-exposure image of the membrane stained with an antibody (Santa Cruz H-62) that recognizes both Darpp-32 and t-Darpp. The middle panel is a long exposure of the same membrane and the bottom image shows the levels of actin as a loading control. Numbers below the actin image indicate the ratio of Darpp-32 to t-Darpp signal for each lane in the middle panel, as determined by densitometric scanning. Clones shown in this figure and additional BT/Her^R^ clones have been analyzed multiple times by Western analysis, always with very high t-Darpp expression and low or undetectable expression of Darpp-32.

PPP1R1B encodes two transcription variants, Darpp-32 and t-Darpp [34], both of which hybridize to the same probe set on the Affymetrix GeneChip Human Genome U133 plus v2.0 array used for this analysis. To determine if one or both transcription variants encoded by the PPP1R1B gene were up-regulated in BT/Her^R^1.0 clones, we extracted RNAs from parent BT474 cells and two BT/Her^R^1.0 clones and measured Darpp-32 and t-Darpp mRNA levels in these cells. BT/Her^R^0.2 clones, which did not have up-regulated PPP1R1B in the microarray analysis (data not shown), were also analyzed in this experiment. Quantitative real-time RT-PCR revealed an increase in the t-Darpp mRNA levels in BT/Her^R^ cells relative to parent BT474 cells, including a slight increase in the BT/Her^R^0.2 clones (Fig. 1A). In contrast, no significant up-regulation of Darpp-32 mRNA levels was observed in BT/Her^R^ cells vs. BT474 cells. Consistent with the quantitative RT-PCR data, Western analysis confirmed that t-Darpp protein expression was up-regulated in all BT/Her^R^ clones analyzed, whereas BT/Her^R^ clones and their parent BT474 cells expressed similarly low levels of Darpp-32 protein (Fig. 1B). A longer exposure of the gel revealed detectable expression of the full-length Darpp-32 protein and perhaps a modest elevation of this protein in BT/Her^R^ cells, relative to BT474 cells (Fig. 1B), but t-Darpp was clearly the predominant variant overexpressed in BT/Her^R^ cells. This might suggest an overall activation of the PPP1R1B locus in these cells but preferential transcription from the downstream start site controlling t-Darpp mRNA expression. Since the protein data are not absolutely correlated with the mRNA data in Fig. 1A, there might also be an element of translational or post-translational control over Darpp-32/t-Darpp expression that is currently not understood. Further analysis of the PPP1R1B locus will be required to clarify the mechanisms regulating expression at the mRNA and protein levels.

### Overexpression of t-Darpp confers Herceptin resistance

**Figure 2:**
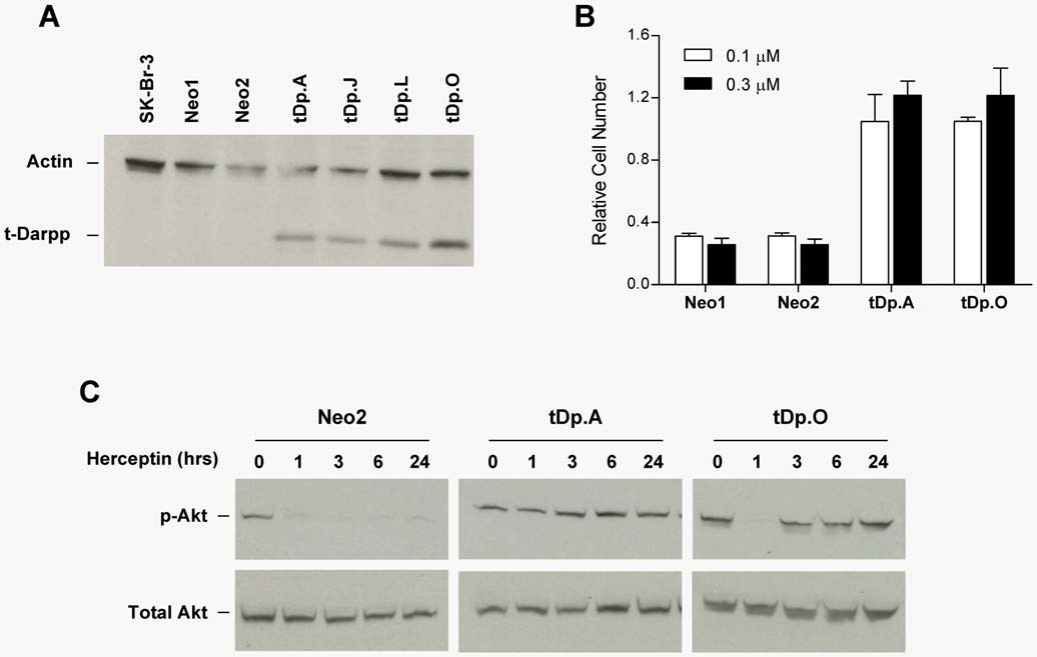
Overexpression of t-Darpp confers Herceptin resistance. (A) SK-Br-3 cells transfected with an empty pcDNA3.0/Neo vector (Neo1 and Neo2) or with a pcDNA3.0/t-Darpp vector (tDp.A, J, L, and O) were analyzed by Western hybridization using the anti-Darpp-32 antibody (H-62) to determine expression of t-Darpp in the transfected cells. (B) Growth of the indicated cell lines was analyzed in the presence or absence of 0.1 µM or 0.3 µM Herceptin, as indicated, for 7 days using a Sulforhodamine B (SRB) assay. Shown are the absorbances of the cells (average of quadruplicate platings, +S.D.) incubated in the presence of Herceptin, normalized to the same cells grown in the absence of Herceptin. (C) Western hybridization was used to measure total Akt and phospho-Akt levels in the indicated clones at various times after the addition of 0.2 µM Herceptin. This experiment was performed twice on the clones shown in this figure and once on an additional clone not shown. Similar results were obtained in both experiments, although clone tDp.A also showed suppression of phospho-Akt levels at the 1 hr time point in the other experiment, with full recovery by 2 hrs (see text).

The purpose of the current study was to determine the phenotypic consequence of activating expression from the PPP1R1B locus. Since t-Darpp was the predominant form of the protein being expressed, we first asked if enhanced expression of t-Darpp is sufficient for conferring Herceptin resistance. We transfected a Her2-positive, Herceptin-sensitive human breast cancer cell line, SK-Br-3, with a cloned human t-Darpp cDNA as described in Materials and Methods. Independent clones stably transfected with either the empty vector (SK.Neo) or the t-Darpp vector (SK.tDp) were isolated. Western analysis confirmed that parent SK-Br-3 cells and SK.Neo clones expressed no detectable levels of t-Darpp or Darpp-32, whereas SK.tDp clones expressed significant levels of t-Darpp protein (Fig. 2A). Herceptin inhibited the growth of the parent SK-Br-3 cells and SK.Neo clones but had no significant effect on the growth of SK.tDp clones (Fig. 2B).

As a further test of the role of t-Darpp in Herceptin resistance, we measured phospho-Akt levels in SK.tDp and SK.Neo clones in the presence and absence of Herceptin. Herceptin caused an almost complete dephosphorylation of Akt in SK.Neo clones, whereas SK.tDp clones were able to maintain a significant level of intracellular phospho-Akt in the presence of Herceptin (Fig. 2C). Interestingly, we observed a reduction in phospho-Akt levels in some SK.tDp clones at 1 hour after the addition of Herceptin, followed by quick restoration of phospho-Akt by 2–3 hours in the sustained presence of Herceptin (Fig. 2C and data not shown), although this result was not reproducible in all clones. Although we do not know the reason for the transient loss of phospho-Akt in some SK.tDp clones, it suggests that Her2's immediate response to Herceptin remains intact in at least some cell lines that overexpress t-Darpp.

### Darpp-32 reverses Herceptin resistance conferred by t-Darpp

Although t-Darpp expression has been associated with malignant transformation and drug resistance, its exact mechanism of action is not known. The full-length Darpp-32, on the other hand, has been extensively studied in neural systems [29] but its possible function in malignancy is not known. We wanted to determine the effect of expressing high levels of Darpp-32 in the presence and absence of its truncated variant, t-Darpp. We transfected SK-Br-3 cells with the pcDNA3.1/Darpp-32 vector as described in Materials and Methods and tried to select clones stably expressing the full-length protein. Transfected clones grew poorly, however, and none with confirmed Darpp-32 expression survived long-term culturing. Out of a combined total of about 50 Zeocin-selected clones isolated from several independent transfection experiments, only 12 were able to grow as cultures and none of those expressed detectable levels of Darpp-32 protein.

**Figure 3:**
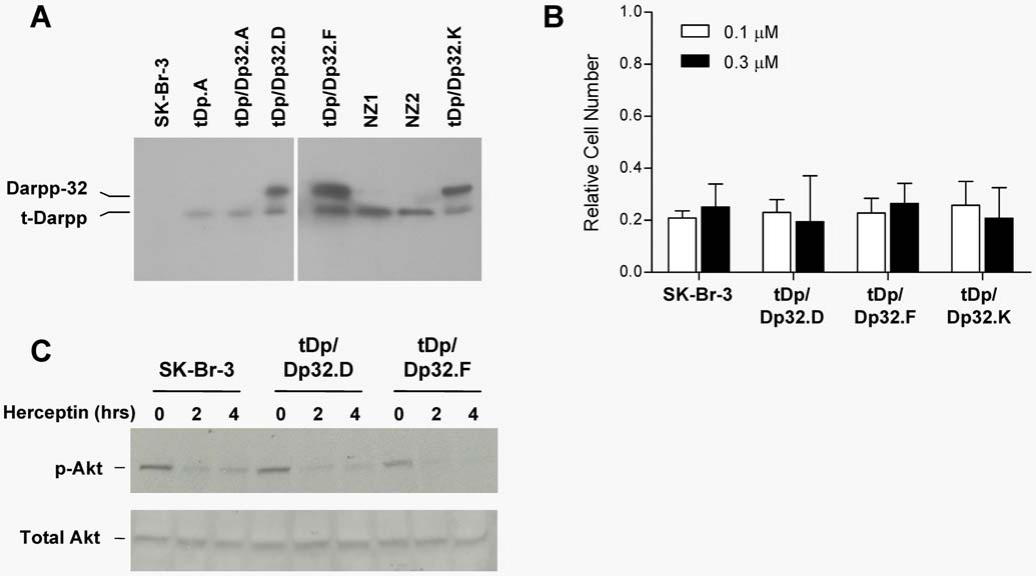
Full-length Darpp-32 reverses Herceptin resistance conferred by t-Darpp. (A) The tDp.A clone was transfected with an empty pcDNA3.1/Zeo vector (NZ1 and NZ2) or with the pcDNA3.1/Darpp-32 vector (tDp/Dp32.A, D, F, and K). Expression of t-Darpp and Darpp-32 in these cell lines and parent SK-Br-3 cells was analyzed by Western hybridization using the H-62 anti-Darpp-32 antibody. (B) Growth of SK-Br-3, tDp.A, and tDp/Dp32.D, F, and K was analyzed in the presence and absence of Herceptin (0.1 µM or 0.3 µM) for 7 days. Shown are the absorbances (average of triplicate wells, +S.D.) for cells incubated in the presence of Herceptin, normalized to the same cells grown in the absence of Herceptin. (C) Total and phospho-Akt levels in SK-Br-3, tDp/Dp32.D, and tDp/Dp32.F clones at the indicated times (hrs) after the addition of Herceptin, analyzed by Western hybridization. This experiment was performed three times, twice with 1.0 µM Herceptin, once with 0.2 µM Herceptin. We show the results with 0.2 µM Herceptin, representative of all three experiments showing loss of phospho-Akt signal by 4 hours.

These results suggested that Darpp-32 overexpression might be detrimental to cell proliferation and/or survival in SK-Br-3 cells. If true, then Darpp-32 and t-Darpp would appear to have opposite effects on cell growth. If Darpp-32 and t-Darpp do functionally antagonize each other, we reasoned that enhanced expression of Darpp-32 might reverse the Herceptin resistance phenotype conferred by t-Darpp overexpression. To test this hypothesis, we transfected Darpp-32 cDNA into the Herceptin-resistant SK.tDp.A clone and selected for zeocin-resistant clones. Out of 11 surviving clones tested, six expressed full-length Darpp-32 in addition to t-Darpp that was already expressed in these cells (Fig. 3A and data not shown). Thus, the presence of t-Darpp appeared to create a permissive environment for enhanced Darpp-32 expression that otherwise could not be achieved. We next tested these cells for their response to Herceptin. The SK.tDp/Dp32 clones that expressed both t-Darpp and Darpp-32 were sensitive to Herceptin-induced growth arrest (Fig. 3B). Moreover, Herceptin caused nearly a complete dephosphorylation of Akt in SK.tDp/Dp32 clones, similar to that observed in the parent SK-Br-3 cells (Fig. 3C) and SK.Neo clones (not shown).

### t-Darpp and Darpp-32 have antagonistic effects on CRE binding activities

**Figure 4:**
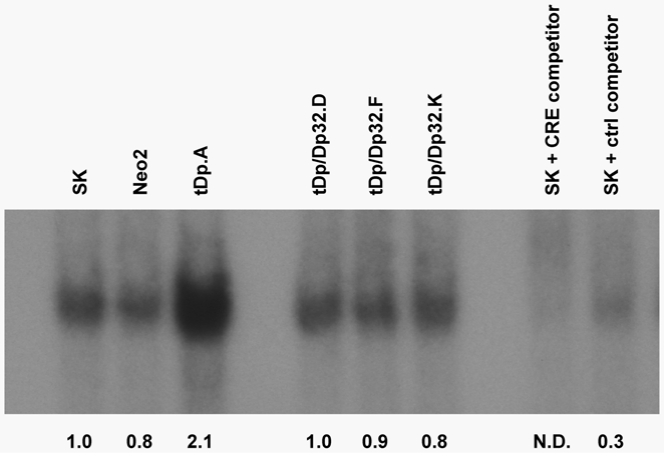
t-Darpp and Darpp-32 have antagonistic effects on CRE binding activity. Nuclear CRE binding activity in parent SK-Br-3 cells (SK) and the indicated transfected clones was analyzed by EMSA, as described in Materials and Methods. Quantification of band density, relative to the SK lane, is shown below each lane. The experiment was performed twice and the relative band densities for the other experiment were 1.9 for tDp.A and 1.1, 0.5 and 0.4 for the three tDp/Dp32 clones, respectively.

Darpp-32 is a substrate for and negative regulator of PKA. Although the relationship between t-Darpp and PKA has yet to be elucidated, El-Rifai's group recently reported that exogenous expression of t-Darpp in AGS gastric carcinoma cells leads to increased phosphorylation of CREB, a PKA substrate, and increased CRE binding activity [32]. One explanation for this observation may be that, opposite to Darpp-32, t-Darpp acts as an activator of PKA or has a dominant-negative effect on Darpp-32's inhibitory activity. To determine the effect of t-Darpp and Darpp-32 overexpression on PKA activity, we performed an electrophoretic mobility shift assay on nuclear extracts derived from SK.tDp.A cells and three independent SK.tDp/Dp32 clones, using a consensus CRE sequence as probe. Parent SK-Br-3 cells and a SK.Neo clone were used as controls. The nuclear extract from clone SK.tDp.A that expressed t-Darpp alone contained higher CRE binding activity than the controls (Fig. 4). Conversely, nuclear extracts derived from three independent SK.tDp/Dp32 clones that expressed both t-Darpp and Darpp-32 had reduced CRE binding activity relative to the SK.tDp.A clone from which they were derived. Although we do not know if the observed changes in CRE binding activity due to exogenous expression of t-Darpp alone or co-expression of t-Darpp and Darpp-32 were mediated directly through the PKA pathway, this result is certainly compatible with an effect of these proteins on PKA signaling.

## Discussion

We have previously reported on the isolation and initial characterization of BT/Her^R^1.0 cells that are highly resistant to Herceptin [8]. Those cells were derived by culturing the Her2-dependent, Herceptin-sensitive BT474 human breast cancer cells in the presence of 1.0 µM Herceptin for 5–6 months. Gene profiling was subsequently performed to determine the full complement of gene expression changes in BT/Her^R^ clones. That analysis revealed changes in a number of genes that intersect with the PKA signaling pathway as either direct or indirect regulators of PKA activity. These include down-regulation of the gene for PKA-RIIα, a PKA regulatory subunit; down-regulation of PKIγ, an endogenous inhibitor of PKA [27]; upregulation of PPP1R1B, which codes for Darpp-32 and its transcriptional variant t-Darpp [29], [34]; and down-regulation of PPP1R3C, whose gene product has multiple functions including promotion of protein phosphatase-1 (PP-1) activity and modulation of Darpp-32 function [28]. The net effect of these changes is 2- to 4-fold activation of PKA signaling activity in BT/Her^R^ cells (manuscript submitted).

In the current report, we demonstrate that it is the amino-terminal truncated protein, t-Darpp, that was predominantly overexpressed in BT/Her^R^ cells selected for Herceptin resistance (Fig. 1) and that exogenous overexpression of t-Darpp was sufficient for conferring Herceptin resistance on Her2-positive SK-Br-3 cells (Fig. 2). These results are consistent with the recent report from Belkhiri *et al*. using independently derived Herceptin-resistant cell lines and transfected cells [31]. Hamel *et al*. also demonstrate that t-Darpp overexpression can confer resistance [35]. We go on to demonstrate that high expression of the full-length protein, Darpp-32, was able to reverse t-Darpp's effects on Herceptin-resistant cell growth and phospho-Akt levels (Fig. 3). In fact, overexpression of Darpp-32 alone appeared to be deleterious to cell survival, since we were never able to obtain long-term cultures of SK-Br-3 cells that overexpress Darpp-32. These data suggest that Darpp-32 and t-Darpp have antagonistic effects on cell growth and/or survival.

In addition to conferring Herceptin resistance, t-Darpp is also reported to confer resistance to drug-induced apoptosis, apparently via a mechanism that involves CREB activation [32]. We also show a relationship between t-Darpp overexpression and CREB activation and this was reversed by co-expression of Darpp-32 (Fig. 4). Although not definitive, the most likely means by which CREB is influenced by t-Darpp/Darpp-32 is via the PKA signaling pathway. This would be consistent with the overall implication of PKA regulatory proteins in Herceptin resistance discussed earlier and the known function of Darpp-32 as a PKA inhibitor [29], but the mechanism by which t-Darpp might influence this pathway is not known. Because the full-length Darpp-32 is both a substrate for phosphorylation by PKA (at Thr-34) and a feedback negative regulator of PKA, it is interesting to speculate that t-Darpp might function by antagonizing Darpp-32's feedback inhibition of PKA, thereby potentiating PKA signaling. The downstream effects of PKA signaling include phosphorylation and activation of full-length Darpp-32 as a PP-1 inhibitor [29]. Notably, the Thr-34 phosphorylation site and PP-1 binding domain are missing from t-Darpp, suggesting that its overexpression should not have a direct effect on PP-1 activity.

There could be multiple downstream effects of PP-1 inhibition and/or PKA activation, including the sustained phosphorylation and constitutive activation of Akt that is observed in Herceptin resistant cells. As noted earlier, Belkhiri *et al*. found that t-Darpp is anti-apoptotic and promotes resistance to cytotoxic agents, additional effects that could be mediated through PP-1 or more directly through PKA [31], [32]. One final observation from our reported microarray data is the down-regulation of PPP1R3C in BT/Her^R^1.0 clones. The corresponding gene product, PTG, is a scaffolding protein that can stimulate PP-1 activity and also has been reported to interfere with Darpp-32's inhibition of PP-1 [28]. Thus, down-regulation of PTG would be consistent with a mechanism that results in overall inhibition of PP-1 and sustained Akt phosphorylation.

**Figure 5:**
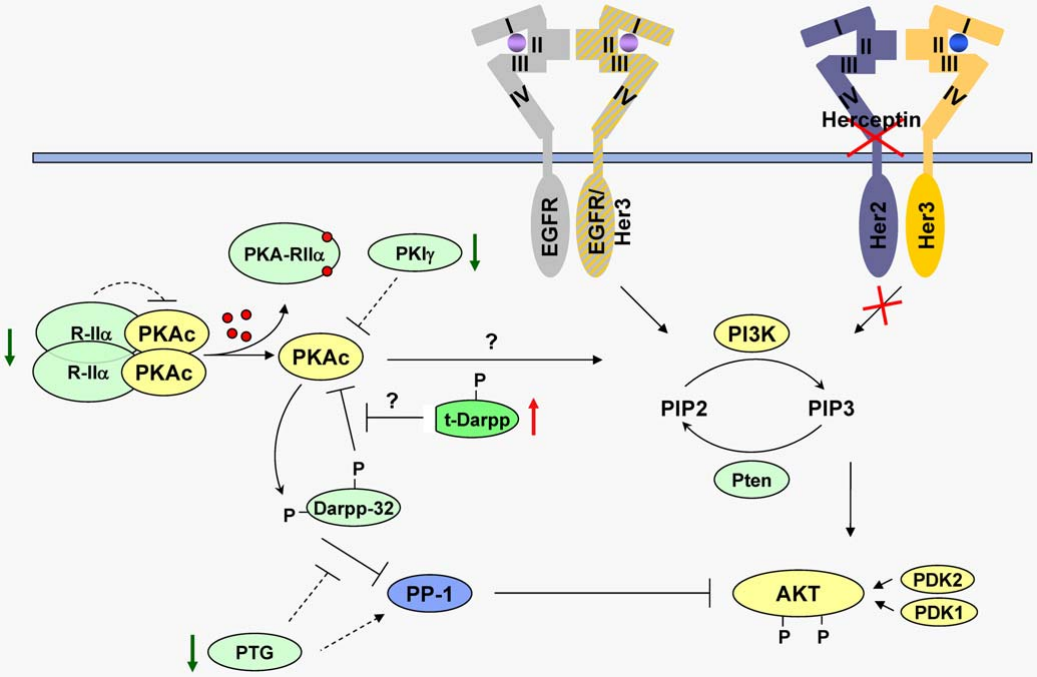
A working model of Herceptin resistance in BT/Her^R^ cells. Several intracellular changes, including down-regulation of PKA-RIIα, PKIγ and PTG (green arrows) and up-regulation of t-Darpp (red arrow) work coordinately to enhance PKA activity. PKA, in turn, either activates the PI3K/Akt pathway (possibly through EGFR or the p85 regulatory subunit of PI3K) or promotes sustained phospho-Akt levels by activating the PP-1 inhibitory activity of Darpp-32. This activity is stimulated via a phosphorylation event at Thr-34, which is absent from t-Darpp. A second phosphorylation event, at Thr-75, activates Darpp-32 as a PKA inhibitor. We speculate that t-Darpp may interfere with this PKA inhibition via a dominant negative mechanism. Dashed lines indicate activities that are down-regulated in BT/Her^R^ cells relative to BT474 cells. Question marks indicate hypothetical pathways that are activated in BT/Her^R^ cells. All other pathways represent well established activities associated with the indicated proteins and enzymes. Abbreviations not already cited: PDK1/2, 3-phosphoinositide-dependent kinase-1/2; Pten, phosphatase and tensin homologue deleted in chromosome 10; PIP2, phosphatidylinositol-4,5-bisphosphate; PIP3, phosphatidylinositol-3,4,5-trisphosphate.

Based on the findings in the current report, our previous observations on the role of PKA and its regulatory proteins in Herceptin resistance, and the work of Belkhiri *et al*., we propose a working model in which down-regulation of PKA-RIIα, PKIγ and PTG and up-regulation of t-Darpp work in concert to enhance PKA enzyme activity and inhibit PP-1, thereby allowing sustained activation of the PI3K/Akt pathway in the presence of Herceptin, and promoting cell growth and survival. This model is illustrated in Fig. 5. Additional direct or indirect effects of t-Darpp overexpression on Her2 or Akt or other downstream effects of PKA dysregulation, as suggested by Belkhiri *et al*. [31], might also contribute to the resistance phenotype. It is worth noting that this same group has not reported the anti-resistance effects of Darpp-32 overexpression that we demonstrate here. In fact, those authors claim that Darpp-32 is able to confer resistance to chemotherapy-induced apoptosis in a colon cancer cell line (RKO) and a gastric cancer cell line (AGS) [33]. Interestingly, Darpp-32 (but not t-Darpp) overexpression results in increased basal apoptosis rates in AGS cells and the protection against drug-mediated apoptosis is more pronounced with t-Darpp overexpression than with Darpp-32 overexpression in those cells. This is especially true for ceramide, a drug that activates protein phosphatases such as PP-1 and PP2A, thus indirectly inhibiting Akt signaling. Hamel *et al.* also suggest that Darpp-32 is able to confer resistance to Herceptin [35], but there is no direct demonstration of Darpp-32 expression in cells analyzed for the Herceptin phenotype in that study. The possible conflicts or overlaps between our current observations and those of Belkhiri *et al*. and Hamel *et al*. still need to be resolved, but they could be due to different cell lines used, different levels of exogenous gene expression, or other factors affecting the intracellular function of Darpp-32.

There could be a broader role for t-Darpp and/or Darpp-32 in malignancy, beyond an involvement in drug resistance. A protein(s) cross-reacting with Darpp-32 antibody and mRNA corresponding to Darpp-32 and t-Darpp are detected in a variety of adenocarcinomas, including breast cancers [30,33,34]. Using antibodies that detect either Darpp-32 alone or both Darpp-32 and t-Darpp, we have found frequent high-level expression of one or both proteins in both Her2-positive and Her2-negative breast cancers (unpublished observations). Work in the current report would suggest that it is t-Darpp, or perhaps the ratio between t-Darpp and Darpp-32, that is critical for both malignant growth and clinical outcome of these cancers, but further analysis will be required to determine if this is the case. If it is, then t-Darpp (or, more generally, the PKA pathway) could become a new target for therapy and/or a biomarker for response to drug therapy. Moreover, it will be interesting to study the mechanism by which cells regulate expression from the upstream (Darpp-32) and downstream (t-Darpp) transcriptional start sites in the PPP1R1B gene, since shifting the balance towards the t-Darpp start site could have profound effects on both malignancy and drug resistance.
